# Accounting for a Quantitative Trait Locus for Plasma Triglyceride Levels: Utilization of Variants in Multiple Genes

**DOI:** 10.1371/journal.pone.0034614

**Published:** 2012-04-02

**Authors:** Lisa J. Martin, Ahmed H. Kissebah, Michael Olivier

**Affiliations:** 1 Divisions of Human Genetics and Biostatistics and Epidemiology, Cincinnati Children's Hospital Medical Center, Cincinnati, Ohio, United States of America; 2 Department of Pediatrics, University of Cincinnati School of Medicine, Cincinnati, Ohio, United States of America; 3 Human and Molecular Genetics Center, Medical College of Wisconsin, Milwaukee, Wisconsin, United States of America; 4 Department of Medicine, Medical College of Wisconsin, Milwaukee, Wisconsin, United States of America; 5 Biotechnology and Bioengineering Center, Medical College of Wisconsin, Milwaukee, Wisconsin, United States of America; 6 Department of Physiology, Medical College of Wisconsin, Milwaukee, Wisconsin, United States of America; Universite de Montreal, Canada

## Abstract

**Background:**

For decades, research efforts have tried to uncover the underlying genetic basis of human susceptibility to a variety of diseases. Linkage studies have resulted in highly replicated findings and helped identify quantitative trait loci (QTL) for many complex traits; however identification of specific alleles accounting for linkage remains elusive. The purpose of this study was to determine whether with a sufficient number of variants a linkage signal can be fully explained.

**Method:**

We used comprehensive fine-mapping using a dense set of single nucleotide polymorphisms (SNPs) across the entire quantitative trait locus (QTL) on human chromosome 7q36 linked to plasma triglyceride levels. Analyses included measured genotype and combined linkage association analyses.

**Results:**

Screening this linkage region, we found an over representation of nominally significant associations in five genes *(MLL3, DPP6, PAXIP1, HTR5A, INSIG1)*. However, no single genetic variant was sufficient to account for the linkage. On the other hand, multiple variants capturing the variation in these five genes did account for the linkage at this locus. Permutation analyses suggested that this reduction in LOD score was unlikely to have occurred by chance (p = 0.008).

**Discussion:**

With recent findings, it has become clear that most complex traits are influenced by a large number of genetic variants each contributing only a small percentage to the overall phenotype. We found that with a sufficient number of variants, the linkage can be fully explained. The results from this analysis suggest that perhaps the failure to identify causal variants for linkage peaks may be due to multiple variants under the linkage peak with small individual effect, rather than a single variant of large effect.

## Introduction

For decades, research efforts have tried to uncover the underlying genetic basis of human susceptibility to a variety of diseases. While our efforts untangling the basis of monogenic disorders have been highly successful [1], [2], [3], advances in the analysis of complex diseases such as diabetes and obesity, or quantitative traits such as blood lipid levels have been slower [4], [5]. The challenge for complex disease is that individual variants contribute little to the overall genetic predisposition. Indeed, recent findings from meta-analyses of genome-wide association studies suggest that even these large-scale efforts so far can only identify variants that together explain 5–15% of the genetic basis of a trait [6], [7], [8], [9]. This suggests that our current analysis approaches and research efforts, including genome-wide association studies, uncover novel genes contributing to disease susceptibility, but still leave large numbers of genes and variants to be uncovered that contribute to the genetic disease risk in the general population [10].

Linkage studies have resulted in highly replicated findings and helped identify quantitative trait loci (QTL) for many complex traits; however identification of specific alleles accounting for the linkage peak has remained elusive [11], [12], [13], [14]. In most follow-up analyses, the assumption has been that single variants (or a limited number of variants) are responsible for the observed linkage, and have significant individual effects. Based on the limited success in finding causal variants, this may be a flawed assumption. To date, numerous variants for a variety of complex traits have been identified in linkage regions such as chromosome 20q for diabetes, but only a small fraction of the total linkage across the region is explained by the identified variants and haplotypes [15]. Therefore, alternative approaches may be needed to comprehensively investigate QTL, and these may require a revised hypothesis about the causality of the observed linkage.

Thus, the purpose of this study was to determine whether with a sufficient number of variants (rather than individual variants) a linkage signal can be fully explained. To accomplish this goal, we analyzed a quantitative trait locus (QTL) on human chromosome 7q36 linked to plasma triglyceride levels (LOD = 3.7) [16], which has been replicated in other studies [17], [18], [19], [20]. To interrogate this genomic interval of approximately 5 Mb, we used comprehensive fine-mapping using a dense set of single nucleotide polymorphisms (SNPs) across the entire QTL interval. We then sought to determine if the linkage evidence can be fully explained using multiple variants.

## Methods

### Study cohort

The Metabolic Risk Complications of Obesity Genes (MRC-OB) project was established in 1994 to identify the genetic determinants of the metabolic syndrome and its metabolic abnormalities [21]. Participant recruitment and individual phenotyping have been described in detail previously [16], [21]. Briefly, families with at least two obese siblings [body mass index (BMI)>30], the availability of one (preferably both) parent(s), and one or more never obese sibling(s) (BMI<27) were recruited from the TOPS (Take Off Pounds Sensibly, Inc.) membership in 10 Midwestern U.S. states. Health information of all participants was obtained by a questionnaire, which included asthma, kidney or liver disease, hypertension, heart disease, stroke, thyroid disorders, diabetes, medications, menopausal status and hormonal therapy, weight history, and smoking history. Individuals were excluded from recruitment with the following conditions: pregnancy, type 1 diabetes, history of cancer, renal or hepatic disease, severe coronary artery disease, substance abuse, corticosteroids or thyroid dosages above replacement dose, history of weight loss of more than 10% in the preceding 12 months, as well as individuals receiving lipid-lowering medications. Phenotypic measurements included height, weight, waist and hip circumferences, and fasting plasma levels of glucose, insulin, total cholesterol, LDL-cholesterol, HDL-cholesterol, and TG. A total of 2209 individuals distributed over 507 families of Northern European descent qualified for the above-mentioned criteria and thus formed the initial study population, of which 1560 individuals from 261 families that contributed most to the linkage were selected for further studies.

All protocols have been approved by the Institutional Review Board of the Medical College of Wisconsin. Details on the cohort used in this study are included in Table 1.

**Table 1:** Descriptive Statistics of the Take Off Pounds Sensibly (TOPS) Cohort and the subsets used for following up linkage analysis.

Trait	Original Linkage Cohort	Follow Up Cohort – linking to 7q36	Individuals with Complete Genotyping on 120 SNPs
N	2007	1235	684
# Families	401	258	225
Family Size (mean (range))	5.0 (2–29)	4.8 (2–27)	3.0 (1–10)
Age (years)	48.12±15.10	47.92±14.80	47.47±14.89
Triglycerides	128.73±81.19	129.57±83.70	130.04±80.45
Ln triglycerides	4.71±0.53	4.71±0.54	4.72±0.54
Sex (% male)	23.5	23.8	21.5
Asthma (% Y)	8.5	9.5	8.8
Menopausal (% Post)	40.0	40.5	42.8
Estrogen Use (% Y)	17.7	18.5	19.7
Ever Smoke (% Y)	22.0	16.0	17.0
Cholesterol/LDL medications	3.5	2.9	2.6
Type 2 Diabetes (% Y)	15.9	16.9	19.4
Diabetes Medications (% Y)	5.2	5.7	6.4

### Initial Linkage Analysis

As previously reported (Sonnenberg et al), 2,209 individuals from 507 families were ascertained for basic anthropomorphic phenotypes, plasma lipid measures, and fasting glucose and insulin levels. A genome-wide linkage scan of these families identified a quantitative trait locus (QTL) on human chromosome 7q36 linked to plasma triglyceride levels (LOD = 3.7). The locus spans approximately 5 Mb of genomic sequence, and contains 18 known genes.

### Genotyping

We selected 1,048 tagSNPs spanning the entire 5 Mb region using the tagger function of Haploview [22]. All SNPs were genotyped on an Affymetrix MegAllele custom-designed 3K array using molecular inversion probe technology, as described previously [23], [24].

### Statistical Analysis

As triglyceride levels are continuously distributed, the data were first examined for deviations from normality. Raw triglyceride levels exhibited an increased number of high values, so the data were natural log (*ln*) transformed. Data were re-examined and observations exceeding 4 standard deviation units were removed as outliers, as described previously [16]. All genetic analyses were performed using the variance components framework and the computer program SOLAR (Texas Biomedical Research Institute, San Antonio, TX) [25].

#### Single SNP Analysis

For each SNP, we used the measured genotype approach to test for association. Briefly, in this approach, the effects of the individual SNP genotypes will be modeled by assigning genotypic values such that the homozygotes will be assigned values of 1 and –1 and the heterozygotes will be assigned 0, providing an additive model [26]. To account for the phenotypic correlation between family members, we used variance components analysis with the SNPs screened individually as covariates. Other covariates for the single SNP models included age, age by sex, sex, age squared, age squared by sex, asthma, menopause status, birth control use, smoking status, lipid lowering medications, and diabetes status. To test the effects of individual SNPs, log likelihoods of models estimating the effect of the SNP are compared to the log likelihoods of models in which the SNP effect is constrained to zero. Under the assumption that trait values in a family follow a multivariate normal distribution, twice the difference in the log likelihoods of these two models is asymptotically distributed as a chi-square with one degree of freedom.

To determine whether any one SNP accounted for the linkage peak, we then performed combined linkage association. In this approach, we evaluated the evidence of linkage after accounting for the SNP. If the SNP accounted for all of the linkage peak, the LOD score would be expected to drop below 0.5 [27]. All combined linkage association models included age, age by sex, sex, age squared, age squared by sex, asthma, menopause status, birth control use, smoking status, lipid lowering medications, and diabetes status as covariates.

#### Multiple SNP Analysis

In order to assess whether clusters of SNPs account for the observed linkage, we first examined the location of all nominally associated SNPs from the initial analysis to identify regions where these associated variants cluster.

To dissect the genetic effects of these cluster regions and to assess the magnitude of effect on TG levels and the originally observed linkage, we selected all SNPs in these cluster regions that were used in the single SNP screen (n = 324), including SNPs regions that showed no evidence of single SNP association. To minimize redundant information, SNPs were eliminated from consideration if they exhibited substantial LD with other SNPs in the analysis (r^2^>0.7). For the sets of SNPs exhibiting substantial LD, SNPs were eliminated on the basis of missing data, such that SNPs with the lowest percentage of missing data were retained in the analysis. Importantly, previous evidence of association was not considered in the reduction of the SNP sets. The selected SNPs were included in a model where each SNP was modeled additively. Importantly, as our goal was not to determine the significance of any specific SNP in the multi-SNP model, all SNPs were forced to be included in the model but significance testing was not performed. Other covariates included age, age by sex, sex, age squared, age squared by sex, asthma, menopause status, birth control use, smoking status, lipid lowering medications, and diabetes status, as these were the covariates included in the initial linkage analysis when the original QTL was identified.

To estimate the impact of incorporating these SNPs, we performed a combined linkage association analysis. Briefly, in this analysis we evaluated the evidence of linkage when all selected SNPs were included in the analysis to a model where the effect of all of the SNPs was constrained to zero. This permitted the comparison of models with the same individuals. We then determined whether the linkage peak was abolished (LOD<0.5) [27]. As the linkage peak may be abolished because of over-parameterizing the model, we then performed 500 permutations where the phenotypes were randomly assigned to individuals. In each permutation, the LOD score was estimated when all SNPs were included in the model. We then counted the number of times that the LOD score was below the 0.5 threshold. The empirical significance is the number of times the LOD was below the threshold plus one divided by the number of permutations performed.

### Results

#### Single SNP Analysis

**Figure 1:**
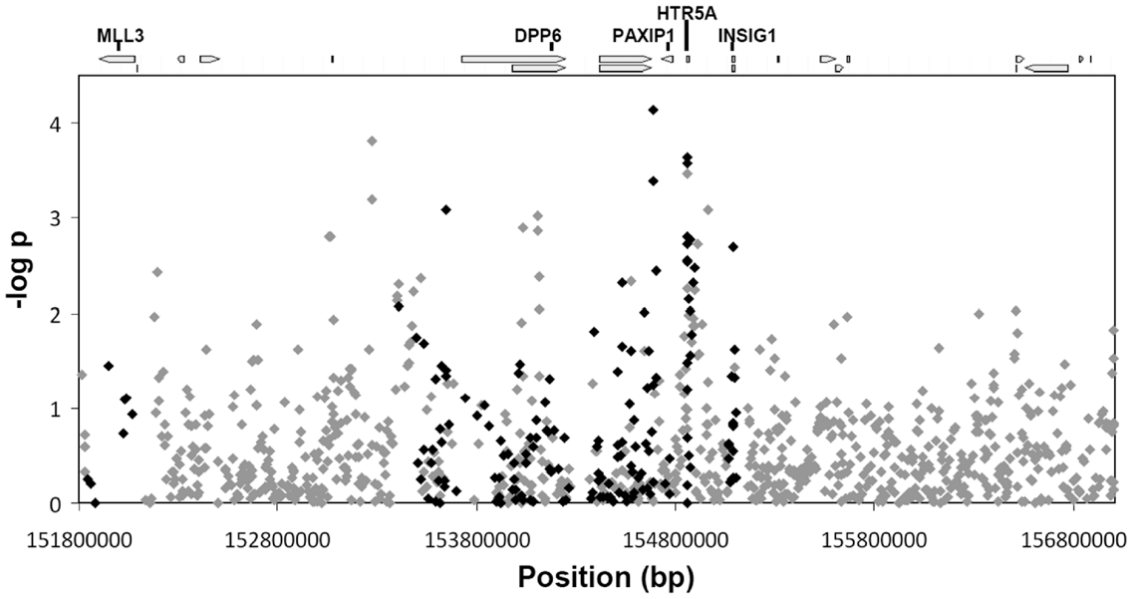
Plot of single SNP association across the QTL interval on human chromosome 7q36. The positions for each SNP are listed on the x-axis, and are based on the genome assembly hg19. SNPs included in the targeted multi-SNP analysis (160 SNPs) are highlighted in back. The bars on the top of the diagram highlight the position of all coding sequences in the interval, the five genes of interest are labeled.

The results for the association analysis of individual SNPs with plasma TG levels are illustrated in Figure 1. Of the 1,048 SNPs, 109 showed association with plasma TG levels at a nominal p-value of less than 0.05. Nine SNPs had p-values of less than 0.001. Of all associated SNPs, 89 SNPs (82%) clustered in regions of high LD around five genes (including 10 kb upstream and downstream of the coding region), MLL3, DPP6, PAXIP1, HTR5A, and INSIG1, suggesting that these gene regions contribute to the altered triglyceride profiles characteristic of the metabolic syndrome in our cohort.

When examining the effect on the LOD score of each of the 109 SNPs which exhibited nominal p-values we found that no one SNP explained the linkage peak (LOD score dropped below 0.05). Indeed, the median value for the proportion LOD decline was 0.03 (interquartile range: 0.002–0.05). Further, only 15 SNPs exhibited LOD drops greater than 0.10. Taken together these data suggest that any one SNP in this region is insufficient to explain the linkage peak.

#### Multiple SNP Analysis

For further analysis, we selected all SNPs (n = 324) surrounding these five genes identified in the single SNP screen. Of these variants, 160 SNPs had low pairwise linkage disequilibrium measures (r^2^<0.7). When all 160 SNPs were included in the analysis model, 684 individuals had complete genotype information for all SNPs and thus entered the analysis. The covariates (including the SNPs) explained 36% of the variance in triglycerides and there was no evidence of any additional genetic effect for the locus on 7q36 (heritability = 0, LOD = 0.05 at the QTL). When restricting the analysis to the same 684 individuals but not including the SNPs as covariates, the remaining covariates explained only 5% of the variation in triglycerides and there was strong evidence of a genetic effect (heritability = 0.26±0.08, LOD = 3.1 at the QTL).

Of the 500 permutations, only 3 achieved LOD scores less than 0.5, resulting in an empirical p-value of 0.008 for the LOD score to fall below 0.5. Further, in the permutations, the LOD score never reached the LOD score obtained with the real data, supporting the significance of these findings.

These data clearly indicate that the observed linkage effect is not due to a single variant. To ensure that a single gene was not driving the analysis, we then split these 160 SNPs into the five genes. Results from the by gene analyses are presented in Table 2. Briefly, the number of SNPs per gene ranged from 5 (PAXIP1) to 111 (DPP6). The proportion of the LOD score decline ranged from 1.15% (PAXIP1) to 81.3% (DPP6). However, the proportion of decline was associated with the number of SNPs included (p = 0.002). As there are a large number of SNPs included from DPP6, we recognize that the proportion of variation explained by this gene is elevated likely due to long range LD with the other candidate genes in this region. Importantly, even including multiple variants from a single gene is not sufficient to obtain evidence for the accounting of the LOD score. Only the combination of multiple SNPs across multiple genes is sufficient to fully explain the observed linkage signal.

**Table 2:** Impact of Adjusting for SNPS by Candidate Gene.

Gene	Number of SNPs	Base LOD	Adjusted LOD	% Decline
MLL3	8	9.14	8.88	2.8
PAXIP1	5	7.96	7.87	1.15
INSIG1	16	6.36	5.23	17.7
HTR5A	20	7.76	5.89	24.1
DPP6	111	5.78	1.08	81.3

### Discussion

With recent findings, it has become clear that most complex traits are influenced by a large number of genetic variants each contributing only a small percentage to the overall phenotype. Thus the question remains for complex traits whether a linkage peak can be explained in entirety with a sufficient number of variants. Using a family based cohort which exhibited linkage for plasma triglycerides to human chromosome 7q36, we have demonstrated that a single genetic variant is insufficient to account for the linkage peak. Further, we demonstrated that using multiple variants capturing the variation in five genes *(MLL3, DPP6, PAXIP1, HTR5A, INSIG1)*. this linkage peak can be explained completely.

In this study, we coupled information from linkage and association to clarify the genetic basis of variation in plasma triglyceride concentrations. A major benefit of using this combined approach is that these methods provide independent information [28] and thus improve power [29]. Therefore, regions where there is already prior evidence of linkage are more likely to contain causal variants than regions which do not. In addition, the false-positive results from linkage can be vetted by requiring that SNPs are not only associated but also explain at least part of the linkage peak because of the independence of the two tests. Indeed, this approach has been used in simulated data to demonstrate its power to detect the functional variants [30], [31].

Using a comprehensive set of 1,048 tagSNPs identified from the publicly available data of the International HapMap Consortium [32], we examined the effect of both individual SNPs and clusters of variants on the observed linkage. Of the 1,048 SNPs, 109 SNPs showed nominal association with plasma TG (p<0.05). Of these SNPs, 89 (82%) clustered around five genes, MLL3, DPP6, PAXIP1, HTR5A, and INSIG1, suggesting that these genes significantly contribute to the altered lipid profiles. Several of these genes are biologically plausible candidates for lipid related phenotypes. INSIG1 has been demonstrated to regulate expression of sterol regulatory element-binding protein target genes and are required for lipogenesis [33]. While MLL3 and PAXIP1 have not been implicated in lipid metabolism, they play a role in PPARγ dependent adipogenesis [34], [35] and activation of PPARγ improves lipid homeostasis [36]. Lastly, HTR5A is involved in central metabolic balancing [37], and many of the serotonin receptor genes have been associated with obesity related traits [38]. DPP6 has not been implicated in lipid metabolism rather seems to be important in neuronal signaling [39], [40].

While these genes are plausible, no single SNP explained a significant fraction of the linkage, suggesting that multiple variants of small individual effect account for the QTL. While the identification of causal variants following an initial linkage study has worked well for Mendelian disorders, the failure to identify a single variant that accounts for a linkage peak for a complex trait consistent with previous fine-mapping studies [11], [12], [13], [14], [41]. A reason for such a failure may be allelic heterogeneity within the locus.

On the other hand, accounting for many variants irrespective of the level of association can account for the linkage peak. One hundred sixty variants (15.3% of the 1048 SNPs tested) randomly selected from these five genes completely account for the linkage on chromosome 7q36. However, as the purpose of this paper was to determine if capturing the variation present in these five genes would be sufficient to explain the linkage, we did not seek to identify the minimum number of variants required to explain the linkage or the specific variants. Given the linkage disequilibrium in this region, it is plausible that the causal variants are not in the 160 variants selected. Future studies will need to examine this locus in more detail to identify the specific variants responsible for the linkage.

These results suggest that a single genetic variant is not likely to be the cause of a linkage signal for a complex trait. Indeed, even in the simple Mendelian inherited diseases such as cystic fibrosis [42] and Holt Oram syndrome [43], [44] exhibit multiple causal variants at a locus. This scenario creates a challenge for gene discovery with association because association is simply looking for whether the phenotype differs by the variant. If multiple variants can all result in the same phenotype, then the power to detect association is small. However, in linkage analysis the assumption is that the region segregates with disease, thus if there are multiple causal variants segregating, evidence of linkage will be present but not evidence of association. Previous studies have failed to find strong relationships between linkage peaks and association peaks [10]. Others have speculated that linkage peaks results from multiple variants [41], [45], [46], [47]; our analysis supports this. Thus, future studies will need to utilize methods designed to assess the impact of multiple variants on a linkage peak such as the methods proposed by Chen and colleagues [41].

In this study, we demonstrate that a single SNP or gene is insufficient to explain the linkage of serum triglycerides on 7q36. Indeed, while we observe a cluster of associated SNPs in five genes, any single SNP or gene fails to completely account for the linkage peak. On the other hand, when we select SNPs across all five genes, evidence of linkage disappears, suggesting that multiple variants in these genes significantly alter plasma triglyceride levels. Our findings suggest that the failure to identify causal variants for linkage peaks may be due to multiple variants under the linkage peak with small individual effect, rather than a single variant of large effect.
